# Alternating consumption of β‐glucan and quercetin reduces mortality in mice with colorectal cancer

**DOI:** 10.1002/fsn3.1187

**Published:** 2019-09-04

**Authors:** Jiamei Qi, Juntong Yu, Yuetong Li, Jianming Luo, Cheng Zhang, Shiyi Ou, Guangwen Zhang, Xinquan Yang, Xichun Peng

**Affiliations:** ^1^ Department of Food Science and Engineering Jinan University Guangzhou China; ^2^ School of Life Sciences Guangzhou University Guangzhou China

**Keywords:** alternate consumption, colonic epithelial gene expression, gut microbiota, quercetin, β‐glucan

## Abstract

The current dietary recommendations for disease prevention and management are scarce and are not well supported. Beta‐glucan or quercetin in a diet can alleviate colorectal cancer (CRC) by regulating the gut microbiota and related genes, but the effects of alternating their consumption for routine ingestion during CRC occurrence remain unknown. This study investigated the effects of alternating the consumption of β‐glucan and quercetin for routine ingestion on CRC development in mice. The mortality rate, colonic length, inflammatory cytokines, gut microbiota, and colonic epithelial gene expression in healthy and CRC mice that consumed normal and alternate diets were compared and studied. The results showed that alternating the consumption of β‐glucan and quercetin (alternating among a β‐glucan diet, a normal diet and a normal diet that was supplemented with quercetin) alleviated colon damage and reduced the mortality rate in CRC mice, with a reduction in mortality of 12.5%. Alternating the consumption of β‐glucan and quercetin significantly decreased the TNF‐α level, increased the relative abundance of *Parabacteroides,* and downregulated three genes (*Hmgcs2, Fabp2,* and *Gpt*) that are associated with inflammation and cancer. Alternating the consumption of some bioactive compounds, such as β‐glucan and quercetin, in food can contribute to human health. This experiment provided some experimental evidence for the dietary recommendations for disease prevention and management.

## 1 INTRODUCTION

Colorectal cancer (CRC) is one of the most common cancers in the world, with 1–2 million new cases diagnosed each year and 700,000 deaths per year (Mármol, Sánchez‐de‐Diego, Pradilla Dieste, Cerrada, & Rodriguez Yoldi, 2017; Stewart & Wild, 2014). One of the most likely causes of CRC is the destruction of the natural relationship between the host and gut microbiota, thus inducing colonic cancer through a chronic inflammatory mechanism (Iebba et al., 2016; Mármol et al., 2017; Serino, Blasco‐Baque, Nicolas, & Burcelin, 2014; Sheflin, Whitney, & Weir, 2014; Tomasello et al., 2014; Wu et al., 2013; Zhang et al., 2015). Colorectal cancer (CRC) is one of the most feared complications of inflammatory bowel disease (IBD) through chronic intestinal inflammation (Eaden, Abrams, & Mayberry, 2001; Jess, Gamborg, Matzen, Munkholm, & Sørensen, 2005; Jess et al., 2012). Inflammatory bowel disease (IBD), including ulcerative colitis (UC) and Crohn's disease (CD), is a chronic intestinal inflammation. Recent studies showed that CRC could be developed by 20% patients with ulcerative colitis (UC) over 30 years (Eaden et al., 2001). Patients with Crohn's disease (CD) are at increased risk of developing CRC, with similar rates of CRC between UC and CD patients (Bernstein, Blanchard, Kliewer, & Wajda, 2001). Although the cause of IBD remains unknown, both genetic background and environmental factors are considered to be related to the pathophysiology and development of IBD (Matsuoka & Kanai, 2015). Among the environmental factors that are associated with IBD, diet plays an important role in modulating the gut microbiome and influencing epigenetic changes (Aleksandrova, Romero‐Mosquera, & Hernandez, 2017).

Diet impacts human health mainly due to the bioactive compounds inside the diet. Many bioactive food compounds, such as β‐glucan and quercetin, have a significant effect on the intestinal environment and then impact human health by regulating the composition of the gut microbiota (Duda‐Chodak, 2012; Laparra & Sanz, 2010; Wang et al., 2016).

Beta‐glucan is a homoglucose polymer that is widely distributed in the cell walls of microorganisms, particularly in those of baker's and brewer's yeast *Saccharomyces cerevisiae*, mushrooms, and cereals (Kittisuban, Ritthiruangdej, & Suphantharika, 2014; Perez‐Quirce, Caballero, Vela, Villanueva, & Ronda, 2018). The gut microbiota can ferment β‐glucan to produce a high proportion of short‐chain fatty acids, such as butyric acid, in the colon, which is considered an important factor to protect the body against colon cancer (Zielke et al., 2017). Some studies also found that β‐glucan can regulate the composition of the microbial community in the intestinal system and reduce the occurrence of colon cancer (Shen, Dang, Dang, Dong, & Hu, 2012; Shen, Lee, et al., 2012; Turunen et al., 2016).

Quercetin is a kind of natural polyhydroxy flavonoid that is found in various foods and plays a role in improving several diseases, such as diabetes, inflammation, and allergies (Hu, Lin, Zheng, & Cheung, 2018; Kee et al., 2016; Lin et al., 2019; Shen, Dang, et al., 2012; Shen, Lee, et al., 2012; Wang, Hu, et al., 2019; Wang, Hu, et al., 2019; Wang, Lam, et al., 2019; Wang, Lam, et al., 2019). Quercetin has a clear antagonistic effect on many kinds of malignant tumors (Kim et al., 2012) and can affect precancerous lesions of colon cancer by inhibiting the release of inflammatory factors (Miyamoto, Yasui, Ohigashi, Tanaka, & Murakami, 2010). It has been reported that quercetin inhibits CRC by inducing cell cycle arrest and apoptosis as well as by enhancing the effects of anticancer drugs (Atashpour et al., 2015; Kee et al., 2016; Zhang et al., 2012). Recently, it has been shown that quercetin may have the ability to modify the gut microbial balance, suggesting that quercetin could act as a prebiotic (Etxeberria et al., 2015; Porras et al., 2017).

Both β‐glucan and quercetin have positive effects on colon cancer and can be consumed via food. However, people do not often simultaneously consume foods containing these compounds, but people often ingest them one after another from different daily diets especially in China. For example, they consume mushrooms that are rich in β‐glucan on one day and enjoy onions that are rich in quercetin on another day. The bioactive compounds are thus discontinuously consumed. The current dietary recommendations for disease prevention and management are scarce and not well supported. The objective of this study was to investigate the effects of alternating the consumption of β‐glucan and quercetin to stimulate routine ingestion during CRC development in mice. This study could provide some experimental evidence for the dietary recommendations for disease prevention and management.

## 2 MATERIALS AND METHODS

### 2.1 Chemicals and dietary formulation

Beta‐glucan was purchased from Henan Baile Chemical Technology Co., China. Quercetin was purchased from TCI Co.. Azoxymethane (AOM) was purchased from Sigma‐Aldrich Corp. Dextran sulfate sodium (DSS, MW 36–50 kDa, colitis grade) was obtained from MP Biomedicals, LLC. Mouse Inflammation ELISA Strip was purchased from Beijing Dako Biotechnology Co., Ltd, and a TIANamp stool DNA kit was purchased from Tiangen Biotech Co. Ltd. An AxyPrep DNA gel extraction kit was purchased from Axygen Biosciences, TRIzol^®^ reagent from Invitrogen, TruSeq TM RNA sample prep pit from Illumina, QuantiFluor™‐ST from Promega, Phusion DNA polymerase from NEB, and ChamQ SYBR Color qPCR Master Mix (2X) from Nanjing Novozan Biotechnology Co., Ltd.

All feeds used in this study were provided by the Guangdong Medical Laboratory Animal Center. The feed was irradiated by Co60 (25 kGy) for sterilization. The formulation of the normal diet was based on No. D12450‐B. The β‐glucan diet was made by replacing 10% of the corn starch in the normal diet with β‐glucan (Table 1) (Liu et al., 2015; Xie et al., 2019).

**Table 1:** The formulation of the two diets (g/kg feed weight)

Raw material	Normal diet	β‐glucan diet
Casein	200	200
l‐cysteine	3	3
Corn starch	315	283.5
β‐Glucan	0	31.5
Maltodextrin	35	35
Sucrose	350	350
Cellulose	50	50
Soybean oil	25	25
Lard	20	20
Minerals	35	35
Vitamins	10	10
Choline	2.5	2.5

### 2.2 Animal, diets, grouping, and sample collection

The animal experiments were approved by the Institutional Animal Care and Use Committee of Jinan University, and all Institutional Animal Care and Use Committee of Jinan University guidelines for the care and use of animals were followed. A total of 48 male 6‐week‐old C57BL/6J mice were purchased from Jinan Pengyue Experimental Animal Breeding Company. They were housed in polypropylene cages at a constant temperature (23 ± 2°C) and with 12 hr of light at the Institute of Laboratory Animal Science at Jinan University. After 10 days of adaption to the normal diet (Luo, Zhang, et al., 2018a), the mice were divided into three groups. The 16 mice in the Con Group were fed the normal diet (Table 1), the 16 mice in the Model Group were fed the normal diet with AOM and DSS included, and the 16 mice in the Norm‐β‐Glu‐Q Group were fed the normal diet, β‐glucan diet and the normal diet supplemented with quercetin by oral administration on different days (one diet per day and every 3 days for a cycle). In this study, the quercetin dosage of 200 mg/kg body weight (Cui, Han, Yang, Sun, & Zhao, 2014; Sriraksa et al., 2012; Wu et al., 2014) was introduced as a supplementation. The Con Group was given distilled water (dH_2_O) during the animal assay, while the Model Group and Norm‐β‐Glu‐Q were given an AOM and DSS solution to cause them to develop colorectal cancer according to our previous research with slight changes (Luo, Zhang, et al., 2018a). Briefly, AOM (12.5 mg/kg) was intraperitoneally injected once on day 0, and the drinking water (dH_2_O) was replaced by a 2% DSS solution on days 8–11, 29–32, and 50–53.

Each group was treated with its corresponding diet for 66 days. Food intake was recorded once a day, and body weight (BW) was recorded every 3 days during the experiment. On day 66, serum was collected from intraorbital blood after allowing the blood to stand overnight at 4°C, followed by centrifugation (1050 *g*). After the animals were sacrificed via cervical dislocation, they were dissected. Cecum contents, colonic epithelial cells, and intact livers and spleens were immediately separately collected. The colon lengths were measured with a vernier caliper.

### 2.3 Disease activity index (DAI) measurement

The methods for the disease activity index (DAI) were based on the previous reports (Xie et al., 2019). The DAI scores of the mice were measured in terms of body weight, fecal property, and hematochezia status, and the total score of the results was divided by 3 to obtain the DAI score (Table 2).

**Table 2:** Scoring system of DAI

Score	Weight loss (%)	Fecal property	Hematochezia status
0	0	Normal	Normal (‐)
1	1–5	Semiloose (+)	Feces with occult blood (+)
2	6–10	Semiloose (++)	Feces with occult blood (++)
3	11–15	Loose (+)	Bloody feces (+)
4	>15	Loose (++)	Bloody feces (++)

Note

The normal stools refer to the granular stool; semiloose stools refer to the paste‐shaped loose stool which do not adhere to the anus or semiformed stool; loose stools refer to the watery stool adhered to the anus. The normal feces, feces with occult blood (+), and feces with occult blood (++) referred to stool without visible blood and showed 3 negative, 1–2 positive, and 3 positive testing results with the Modified EZ Detect Fecal Occult Blood Test Kit, respectively.

The fecal property was classified into normal, semiloose, and loose. Normal stools referred to a granular stool, semiloose stools referred to a paste‐shaped loose stool that did not adhere to the anus or a semiformed stool, and loose stools referred to a watery stool that adhered to the anus. The hematochezia status was classified as normal feces, feces with occult blood, and bloody feces. The normal feces, feces with occult blood (+), and feces with occult blood (++) referred to stools without visible blood that showed 3 negative, 1–2 positive, and 3 positive testing results, respectively, with the Modified EZ Detect Fecal Occult Blood Test Kit.

### 2.4 Preparation of the colon sections

The colons were immobilized by formaldehyde and embedded in paraffin. Then, hematoxylin–eosin (HE) staining was performed (Fan et al., 2005). The pathological sections of the colon were observed with a microscope and evaluated by pathologists.

### 2.5 16S rDNA sequencing of the gut microbiota

A TIANamp stool DNA kit was used to extract the fecal bacterial DNA from six mice in each group according to the manufacturer's instructions. Corresponding to the positions from 338F to 806R in the bacterial 16S rRNA gene are the primers 338F and 806R (ACTCCTACGGGAGGCAGCAG and GGACTACHVGGGTWTCTAAT), and they were used to amplify the V3‐V4 region of the bacterial DNA in each fecal sample by PCR. The PCR was run with a 20 μl reaction system containing 4 μl of 5 × FastPfu buffer, 2 μl of 2.5 mM dNTPs, 0.8 μl of each primer (5 μM), 0.4 μl of FastPfu polymerase, and 10 ng of template DNA. The thermocycler PCR system was used to perform the PCR (ABI GeneAmp^®^ 9700) using the following program: denaturation at 95°C for 3 min, then 27 cycles of amplification including denaturation at 95°C for 30 s, annealing at 55°C for 30 s, and extension at 72°C for 45 s, with a final extension at 72°C for 10 min (Luo, Li, et al., 2018b).

Amplicons were extracted from 2% agarose gels, purified with the AxyPrep DNA gel extraction kit according to the manufacturer's instructions, and quantified using QuantiFluor™ ‐ST. According to the standard protocols, the purified amplicons were pooled in equimolar amounts and paired‐end sequenced (2 × 250) on an Illumina MiSeq platform.

### 2.6 RNA extraction, library preparation, and sequencing

Total RNA was extracted from the colonic epithelial cells using TRIzol reagent according to the manufacturer's instructions. The RNA concentration was determined using a Nanodrop 2000 spectrophotometer (Nanodrop Technologies). The RNA integrity was confirmed by agarose gel electrophoresis, and the RNA integrity number (RIN) was determined by Agilent 2100 (Agilent Technologies). Only high‐quality RNA samples (concentration >50 ng/μL, OD260/280 = 1.8–2.2, OD260/230 ≥ 2.0, RIN ≥ 6.5, 28S:18S ≥ 1.0, >10 μg) were used to construct the sequencing library, and the RNA‐seq transcriptome libraries were prepared with the Illumina TruSeq TM RNA sample prep kit using 5 μg of total RNA. Messenger RNA was isolated according to the polyA selection method by oligo (dT) beads and then fragmented in fragmentation buffer. Then, the synthesized cDNA was subjected to end repair. The library sizes were selected for cDNA target fragments of 200–300 bp on 2% low‐range ultra‐agarose, followed by PCR amplification using Phusion DNA polymerase for 15 PCR cycles. After quantification by TBS380, a paired‐end RNA‐seq sequencing library was sequenced with the Illumina HiSeq 4000 (2 × 150 bp read length).

### 2.7 Read mapping

The raw paired‐end reads were trimmed and quality‐controlled by SeqPrep and Sickle using the default parameters. Then, the clean reads were separately aligned to reference genomes with the orientation mode using Bowtie2 software. The mapping criterion for Bowtie was as follows: sequencing reads should be uniquely matched to the genome, allowing up to two mismatches without insertions or deletions. Then, the gene region was expanded following the depths of the sites. In addition, the whole genome was split into multiple 15k bp windows that shared 5k bp. Newly transcribed regions were defined as more than two consecutive windows without overlapping gene regions where at least two reads were mapped per window in the same orientation.

### 2.8 Differential expression analysis and functional enrichment

To identify the DEGs (differentially expressing genes) between two different samples, the expression level of each transcript was calculated according to the fragments per kilobase of exon per million mapped reads (FRKM) method. RSEM was used to quantify gene abundances (Li & Dewey, 2011). The R statistical package software EdgeR (empirical analysis of digital gene expression in R) was utilized for the differential expression analysis (Robinson, McCarthy, & Smyth, 2010). In addition, a functional enrichment analysis including GO and KEGG was performed to identify which DEGs were significantly enriched in GO terms and metabolic pathways with Bonferroni‐corrected *p*‐values (also named FDR [false discovery rate]) ≤0.05 compared with the whole‐transcriptome background. The GO functional enrichment and KEGG pathway analysis were carried out by GOATOOLS and KOBAS.

The following criteria were developed to screen the genes that were cancer‐related and alternating diet‐related among all the differential genes: (a) genes that were expressed in all samples in each group (FPKM ≥ 1.0); (b) genes that were significantly enriched in KEGG pathways; and (c) genes that should have been filtered from the differential genes.

### 2.9 Statistical analysis

The experimental results were expressed as the mean and standard deviation, and the statistical analysis was completed by SPSS version 20.0. Significant differences were analyzed by a two‐tailed *t* test. *p* < .05 was considered a significant difference. The experimental results were plotted with Origin 8.0.

## 3 RESULTS

### 3.1 Body weight and food intake

**Figure 1:**
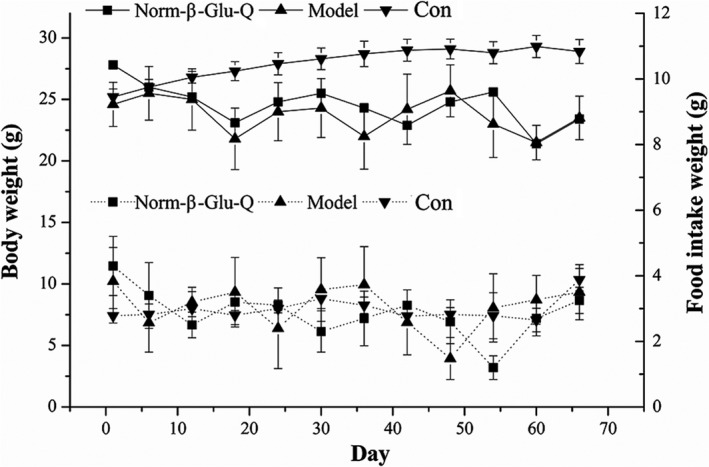
Body weight and feed intake during treatment. Body weight (solid line) and food intake (dashed line) in mice

During the experiment, a threefold reduction in feed consumption was recorded along with severe weight loss in the Model Group and the Norm‐β‐Glu‐Q Group on days 17, 36, and 60 (Figure 1). At the end of the trial, the final BWs of the mice in the Model Group and the Norm‐β‐Glu‐Q Group were lower than their respective initial BWs (*p* > .05), but the final BW in the Con Group was higher than its initial BW (*p* > .05). In addition, the average BW of the mice in the Con Group was higher than that in the Model and Norm‐β‐Glu‐Q Groups (*p* < .05).

### 3.2 Morbidity, mortality, colon length, and DAI score

The prevalence of colon cancer in the Model Group and Norm‐β‐Glu‐Q Group was 100% (Table 3). Compared with the 0% mortality rate in Group Con, up to a 62.5% mortality rate was observed in the Model Group. However, the mortality rate in the Norm‐β‐Glu‐Q Group was found to be 12.5% lower than that in the Model Group (Table 3). The average colon length in the Model Group was significantly shorter than that in the Con Group (*p* < .01), while the average colon length of the Norm‐β‐Glu‐Q Group and Con Group remained similar (*p* ≥ .05; Table 3). The DAI score was significantly higher in both the Model Group and Norm‐β‐Glu‐Q than in the Con Group. However, no significant difference in the DAI score was observed between the Model Group and Norm‐β‐Glu‐Q Group (*p* ≥ .05; Table 3).

**Table 3:** Morbidity, mortality, colon length, and DAI (mean ± standard deviation)

Group	Morbidity (%)	Mortality (%)	Colon length (cm)	DAI
Con	0	0	8.40 ± 0.40	0
Model	100	62.5	7.35 ± 0.44##	2.46 ± 1.12##
Norm‐β‐Glu‐Q	100	50	7.43 ± 0.34	2.39 ± 1.16##

Note

Con: mice without the AOM and DSS treatment and fed a normal diet; Model: mice with the AOM and DSS treatment and fed a normal diet; Norm‐β‐Glu‐Q: mice with the AOM and DSS treatment and alternately fed a β‐glucan diet, a normal diet, and a normal diet that was supplemented with quercetin.

^##^ *p* < .01 when compared with the Con Group.

### 3.3 Colon and colonic pathological sections

**Figure 2:**
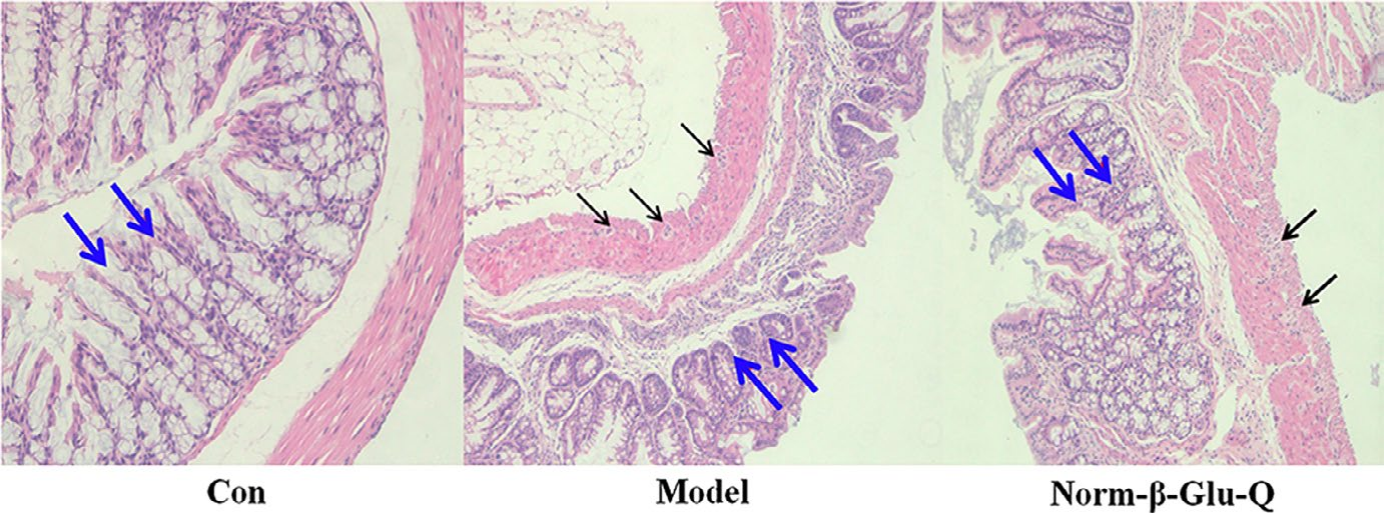
Colonic pathological sections with HE staining (magnification: ×100). Black arrow: tumors; red arrow: massive crypt

Histological studies revealed a normal mucosa layer and villi in the colon of the Con Group but not in the three treated groups. The complete erosion of the epithelium, longer ulcerations, massive crypt loss, and marked lamina propria and submucosa with mixed inflammatory cell infiltration were observed in the Model Group. In addition, tumors occurred in the mucosa layer of the Model Group (Figure 2). The Norm‐β‐Glu‐Q Group showed more normal villi with fewer tumors than the Model Group. The interstitial edema and dilation of vessels were improved, and a general decrease in inflammatory cells (lymphocytes and polynuclear cells) in the lamina propria was observed in the Norm‐β‐Glu‐Q Group (Figure 2).

### 3.4 Inflammatory factor analysis

**Figure 3:**
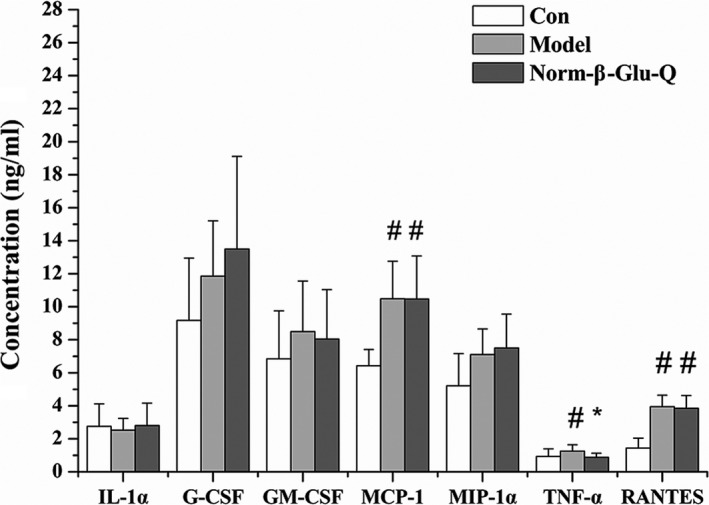
Inflammatory factors of mice. ^#^: *p* < .05 when compared with the Con Group; ^*^: *p* < .05 when compared with the Model Group

The TNF‐α level was significantly higher in the Model Group than in the Con Group and Norm‐β‐Glu‐Q Group (*p* < .05). No significant difference was found between the TNF‐α levels in the Con Group and Norm‐β‐Glu‐Q Group (*p > *.05; Figure 3). The MCP‐1 and RANTES levels in the Model Group were similar to those in the Norm‐β‐Glu‐Q Group (*p > *.05), and they were both significantly higher than those in the Con Group (*p* < .05). The other inflammatory cytokines were not significantly different among the Group Con, Model Group, and Norm‐β‐Glu‐Q Group (*p > *.05; Figure 3).

### 3.5 Overall structure and distribution of the gut microbiota in the mice

A total of 775,663 sequences were detected, with 45,627.24 ± 4,763.92 per sample. The coverage of each group was less than 0.999 (Table 4). The taxonomic diversity and richness of each group at the operational taxonomic unit (OTU) level were analyzed using ecological diversity statistics. The ACE and Chao1 indices in the Model Group were significantly lower than those in the Con Group (*p* < .05), while their Simpson and Shannon indices remained similar (*p* ≥ .05). There were no significant differences in the four α‐diversity indices between the Model Group and Norm‐β‐Glu‐Q Group (*p* ≥ .05; Table 4).

**Table 4:** α‐Diversity indices of the gut microbiota in mice fed different diets with or without the AOM and DSS treatment (mean ± standard deviation)

Group	ACE	Chao1	Simpson	Shannon	Coverage
Con	328.17 ± 24.45	338.23 ± 33.82	0.099 ± 0.045	3.35 ± 0.42	0.9990 ± 0.0001
Model	229.79 ± 64.87^#^	228.99 ± 69.11^#^	0.138 ± 0.111	2.91 ± 0.67	0.9988 ± 0.0003
Norm‐β‐Glu‐Q	246.84 ± 20.86	246.93 ± 27.41	0.080 ± 0.022	3.29 ± 0.24	0.9990 ± 0.0001

Note

*n* = 5 for the Con Group and *n* = 6 for the Model Group and the Norm‐β‐Glu‐Q Group. Con: mice without the AOM and DSS treatment and fed a normal diet; Model: mice with the AOM and DSS treatment and fed a normal diet; Norm‐β‐Glu‐Q: mice with the AOM and DSS treatment and alternately fed a β‐glucan diet, a normal diet, and a normal diet that was supplemented with quercetin. #p < .05 when compared with the Con Group.

**Figure 4:**
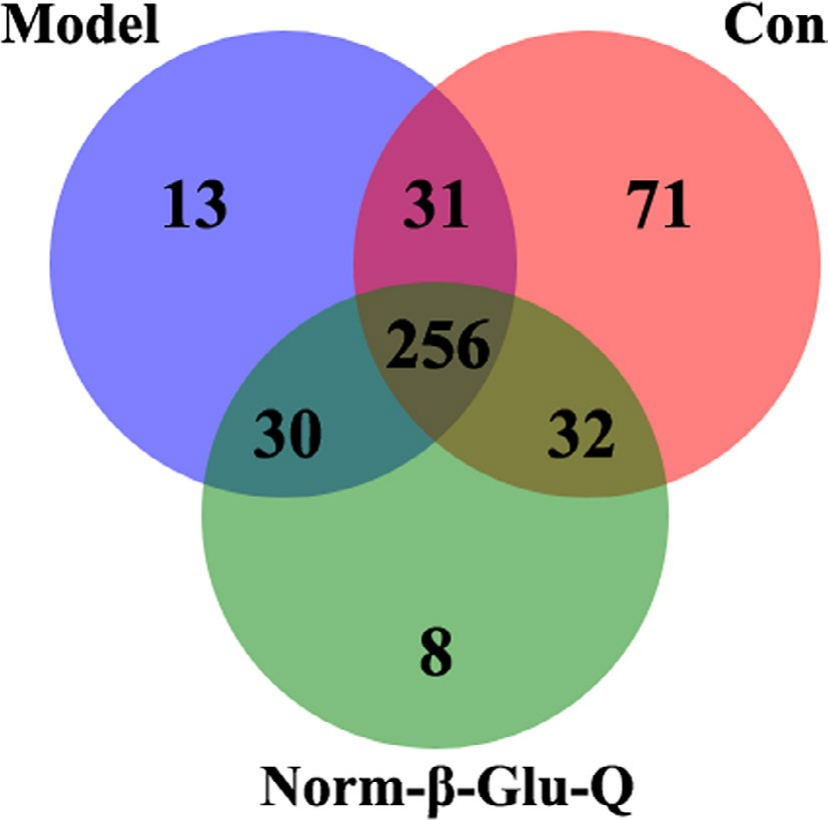
The Venn graph among the Con Group (*n* = 5), Model Group, and Norm‐β‐Glu‐Q Group (*n* = 6 for each group)

The unique and shared OTUs in the various groups are presented in Figure 4. All groups shared 256 OTUs. Additionally, the Norm‐β‐Glu‐Q Group had 8 unique OTUs and shared 30 OTUs only with the Model Group and 32 only with the Con Group. Moreover, the Model Group had 13 unique OTUs, while the Con Group had 71 unique OTUs.

**Figure 5:**
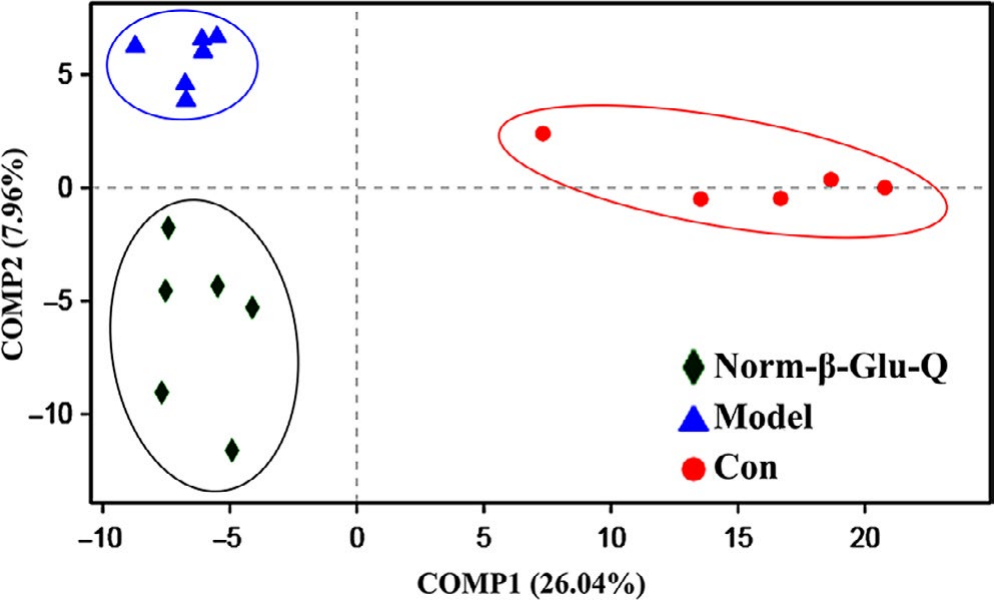
Plots from the partial least‐squares discriminant analysis (PLS‐DA) of gut microbiota among the Con Group (*n* = 5), Model Group, and Norm‐β‐Glu‐Q Group (*n* = 6 for the last two groups)

The distribution of the mouse gut microbiota of the various dietary interventions was presented by a partial least‐squares discriminant analysis (PLS‐DA; Figure 5). The Con Group was located on the right, while the Model Group and Norm‐β‐Glu‐Q Group were located on the left in the PLS‐DA plot. Clear boundaries among the three groups could be obtained according to the plot.

### 3.6 Composition of mouse cecal microbiota at the phylum and genus level

The microbial relative abundance (RA) of each group was altered at the phylum level by the various diets (Table 5). The RA of *Bacteroidetes* in the Con Group was significantly lower (*p* < .01, *p* < .05) than that in the Model Group and Norm‐β‐Glu‐Q Group, while the RA of *Firmicutes* in the Con Group was significantly higher than that in the Norm‐β‐Glu‐Q Group (*p* < .01).

**Table 5:** Relative abundance (%) of mouse cecal microbiota at the phylum level (mean ± standard deviation)

Phylum	Con	Model	Norm‐β‐Glu‐Q
*Bacteroidetes*	12.53 ± 3.29	28.18 ± 14.33#	37.09 ± 9.40##
*Firmicutes*	65.71 ± 5.81	53.35 ± 12.93	47.95 ± 10.56##
*Proteobacteria*	10.09 ± 4.20	7.83 ± 4.84	8.49 ± 4.96
*Verrucomicrobia*	0.00 ± 0.01	0.93 ± 1.27	0.70 ± 0.67
*Deferribacteres*	0.04 ± 0.05	0.86 ± 1.11	0.64 ± 0.42
*Actinobacteria*	11.28 ± 11.91	8.53 ± 5.89	5.02 ± 2.18
Others	0.35 ± 0.16	0.32 ± 0.23	0.10 ± 0.07##, *

Note

*n* = 5 for the Con Group and *n* = 6 for the Model Group and Norm‐β‐Glu‐Q Group. The bacteria at the phylum level with relative abundance less than 1% were classified into “Others”. Con: mice without the AOM and DSS treatment and fed a normal diet; Model: mice with the AOM and DSS treatment and fed a normal diet; Norm‐β‐Glu‐Q: mice with the AOM and DSS treatment and alternately fed a β‐glucan diet, a normal diet, and a normal diet that was supplemented with quercetin.

^#^ *p* < .05 when compared with the Con Group;

^##^ *p* < .01 when compared with the Con Group;

* *p* < .05 when compared with the Model Group.

At the genus level, the RA of *Allobaculum* was significantly lower in both the Con Group (*p* < .05) and Norm‐β‐Glu‐Q Group (*p* ≥ .05) than in the Model Group (Table 6). The RA of *Bacteroides* in the Con Group was similar to that in the Model Group (*p* ≥ .05), and they were both significantly lower than that in the Norm‐β‐Glu‐Q Group (*p* < .05). The Norm‐β‐Glu‐Q Group had the highest RA of *Parabacteroides* (*p* < .05), while the Model Group had the second highest and Con Group had the lowest (*p* < .05). The Model Group had a significantly lower RA of *Roseburia* than the Con Group (*p* < .05).

**Table 6:** Relative abundance (%) of mouse cecal microbiota at the genus level (mean ± standard deviation)

Genus	Norm	Model	Norm‐β‐Glu‐Q
*Faecalibacterium*	18.77 ± 5.68	24.59 ± 17.85	13.49 ± 9.36
*S24‐7* a	9.48 ± 2.51	16.12 ± 7.53	11.66 ± 8.85
*Allobaculum*	7.00 ± 2.10	13.09 ± 4.69#	8.89 ± 6.07
*Erysipelotrichaceae* b	2.03 ± 0.51	3.02 ± 0.93	1.74 ± 1.19
*Bacteroides*	0.50 ± 0.20	9.07 ± 10.6	12.00 ± 1.26#
*Coriobacteriaceae_UCG‐002*	10.51 ± 11.52	6.74 ± 4.83	3.29 ± 2.01
*Desulfovibrio*	9.13 ± 3.61	4.76 ± 4.36	3.33 ± 2.47#
*Parabacteroides*	0.09 ± 0.06	1.42 ± 0.99#	8.63 ± 6.10#, *
*[Ruminococcus]*	0.84 ± 1.86	2.69 ± 2.81	4.09 ± 7.78
*Lachnospiraceae* b	2.58 ± 1.95	1.21 ± 0.85	1.77 ± 0.89
*Lachnospiraceae_NK4A136*	4.00 ± 1.85	0.28 ± 0.24^b^	0.60 ± 0.56^b^
*Akkermansia*	0.00 ± 0.01	0.93 ± 1.27	0.70 ± 0.67#
*Lactobacillus*	0.48 ± 0.75	1.52 ± 1.58	1.69 ± 2.60
*Lachnoclostridium*	2.61 ± 1.87	0.37 ± 0.29#	0.31 ± 0.20#
*Roseburia*	3.03 ± 2.96	0.02 ± 0.02#	0.46 ± 0.53
*Rikenellaceae*	0.12 ± 0.06	0.70 ± 0.81	1.91 ± 3.67
*Escherichia‐Shigella*	0	0.16 ± 0.15#	2.36 ± 4.10
*Alloprevotella*	0.22 ± 0.49	0	1.04 ± 2.51

Note

*n* = 5 for the Con Group and *n* = 6 for the Model Group and Norm‐β‐Glu‐Q Group. Con: mice without the AOM and DSS treatment and fed a normal diet; Model: mice with the AOM and DSS treatment and fed a normal diet; Norm‐β‐Glu‐Q: mice with the AOM and DSS treatment and alternately fed a β‐glucan diet, a normal diet, and a normal diet that was supplemented with quercetin.

a No rank of bacteria.

b Unclassified of bacteria.

^#^ *p* < .05 when compared with the Con Group;

^##^ *p* < .01 when compared with the Con Group;

* *p* < .05 when compared with the Model Group.

### 3.7 Gene expression analysis of mouse colon epithelial cells

**Figure 6:**
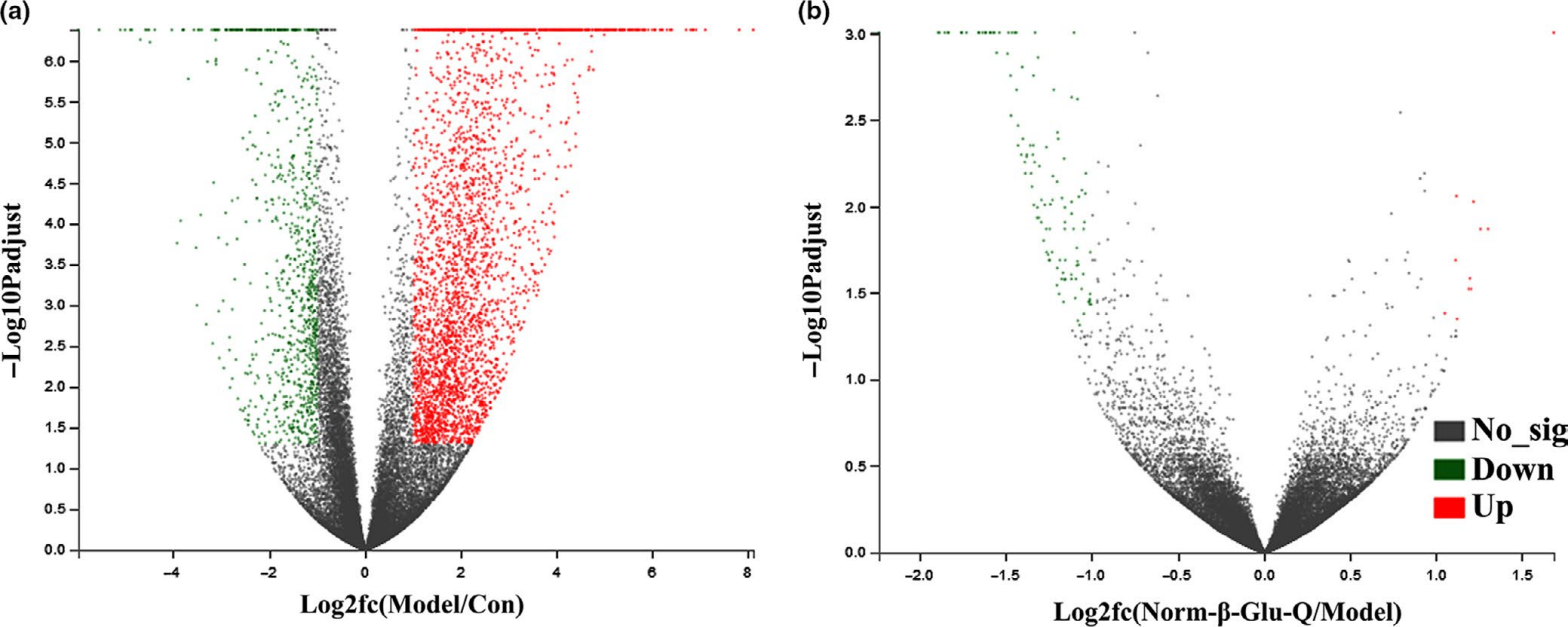
The volcano charts of gene regulation. (a) the differentially expressed genes between the Con Group and Model Group; (b) the differentially expressed genes between the Model Group and Norm‐β‐Glu‐Q Group

**Figure 7:**
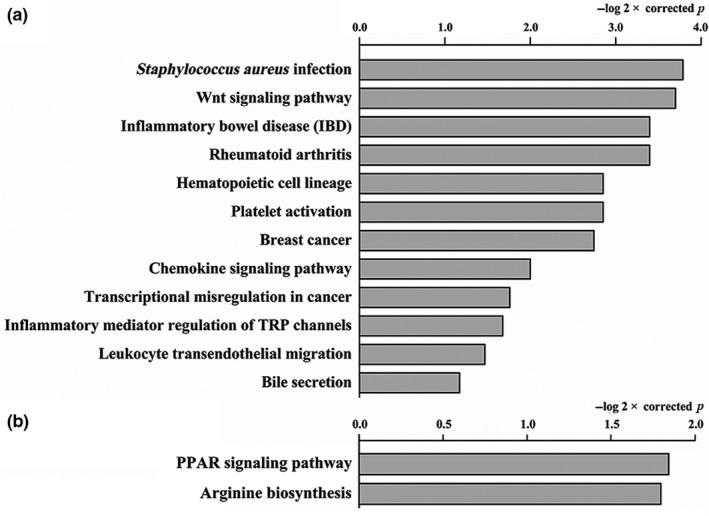
The enrichment of the KEGG pathways. (a) The enrichment of the KEGG pathways that compared the differentially expressed genes in the Con Group and Model Group; (b) The enrichment of the KEGG pathways that compared the differentially expressed genes in the Model Group and Norm‐β‐Glu‐Q Group

In all, 4,011 differentially expressed genes were found between the Model Group and Con Group (*p* < .05). Among them, 3,210 genes were upregulated and 891 genes were downregulated (Figure 6a). The DEGs were significantly enriched in 12 KEGG pathways (Figure 7a; corrected *p* < .05, −log2 × corrected *p* > 1.0) belonging to organismal systems (immune system, sensory system, and digestive system), human disease (infectious diseases: bacterial, immune diseases, cancers: overview, and cancers: specific types), and environmental information processing (signal transduction). Four of the 12 KEGG pathways, including leukocyte transendothelial migration, the chemokine signaling pathway, platelet activation, and hematopoietic cell lineage, were related to the immune system.

The Norm‐β‐Glu‐Q group had 128 differentially expressed genes, including 11 upregulated genes and 117 downregulated genes, when compared with those in the Model Group (*p* < .05; Figure 6b). They were significantly enriched in 2 KEGG pathways (Figure 7b; corrected *p* < .05, −log2 × corrected *p* > 1.0) belonging to metabolism (amino acid metabolism) and organismal systems (endocrine system).

The following criteria were developed to screen the genes that were cancer‐related and alternating diet‐related among all the differential genes: (a) genes that were expressed in all samples in each group (FPKM ≥ 1.0); (b) genes that were significantly enriched in KEGG pathways (Figure 6b); and (c) genes that should be have been filtered from the 128 differential genes.

Finally, three downregulated genes were screened out and listed in Table 7. The KEGG pathways to which the three genes mainly belonged were PPAR signaling pathway related.

**Table 7:** Cancer‐related and alternating diet‐related genes in the Model mice (*n* = 5 for the Con Group and *n* = 6 for the Model Group and Norm‐β‐Glu‐Q Group)

Gene	Log_2_‐fold change (Norm‐β‐Glu‐Q/Model)	Protein	KEGG pathway
*Hmgcs2*	−1.064	HMGCS2	PPAR signaling pathway
*Fabp2*	−1.172	FABP2	PPAR signaling pathway
*Gpt*	−1.092	GPT	PPAR signaling pathway

## 4 DISCUSSION

In this study, mice with CRC were alternately fed a normal diet, a β‐glucan diet and a normal diet that was supplemented with quercetin to investigate whether the alternating consumption of the two functional components impacts the gut microbiota, regulates gene expression in colonic epithelial cells, and hence inhibits the development of CRC. The alternating consumption of β‐glucan and quercetin reduced the mortality rate of mice with CRC. This type of diet showed a significantly higher RA of *Bacteroides* than the other two diets. The induction of CRC in mice resulted in alterations in colonic gene expression. These DEGs were significantly enriched in 12 KEGG pathways, and 4 were immune system‐related. Moreover, the alternating consumption of a normal diet, a β‐glucan diet, and a normal diet that was supplemented with quercetin altered the expression of genes enriched them in 2 KEGG pathways. Among those DEGs, 3 including *Hmgcs2*, *Fabp2,* and *Gpt*, which may contribute to human health, were downregulated due to the alternating consumption of β‐glucan and quercetin.

An AOM and DSS treatment can induce mouse colorectal cancer with 100% morbidity (Thaker, Shaker, Rao, & Ciorba, 2012). Previous reports showed that mice with CRC had symptoms that included a short colon, a high organ coefficient, and even death (Mayol et al., 2006; McKenna et al., 2010). Both β‐glucan and quercetin were confirmed to inhibit colon cancer when they were used alone (Kee et al., 2016; Zielke et al., 2017), and our present results further showed that alternating the consumption of β‐glucan and quercetin reduced the mortality rate by 12%, somewhat alleviating CRC.

Our previous research concluded that β‐glucan reduced colon cancer by regulating the composition of the gut microbial community and improving the intestinal environment but was not related to the inflammatory response (Luo, Zhang, et al., 2018a), while quercetin was reported to inhibit inflammation (Boots, Drent, de Boer, Bast, & Haenen, 2011). Therefore, detecting the levels of inflammatory cytokines, including IL‐1α, MCP‐1, RANTES, G‐CSF, GM‐CSF, MIP1‐α, and TNF‐α, was used to determine the relationships between CRC alleviation and inflammation by the alternating consumption. Among the analyzed cytokines, TNF‐α can induce fibroblasts to secrete protease and destroy the mesothelial layer, which leads to tumor invasion and the regulation of neutrophil gene expression that is related to promoting tumor invasion and migration (Demoulin, Herfs, Delvenne, & Hubert, 2013). Based on our results, the level of TNF‐α was reduced by alternating the consumption of β‐glucan and quercetin, which was probably due to the effect of quercetin since it was reported to be capable of lowering the TNF‐α level (Luo et al., 2017). For the other cytokines, IL‐1α is related to inflammation (Dinarello, Simon, & Van Der Meer, 2012), while MCP‐1 is also a proinflammatory chemokine (Gögebakan et al., 2015). RANTES, which is also known as CCL5, is a chemotactic factor for leukocytes and is important for recruiting T cells, eosinophils, and basophils to inflammatory sites (Matter & Handschin, 2007). Additionally, both G‐CSF and GM‐CSF are highly expressed in colon cancers, while MIP1‐α is higher in tumor tissue (Morris et al., 2014; Tabatabaei et al., 2017; Wang et al., 2014). However, our results showed that no significant reductions were observed for other inflammatory cytokine levels except TNF‐α. This result could be due to the discontinuous consumption of quercetin. Nevertheless, TNF‐α inhibition is one possible mechanism of CRC alleviation from alternating the consumption of β‐glucan and quercetin.

Moreover, the alternating consumption alleviated CRC via changing specific gut bacteria. The genus *Allobaculum* was found to increase the risk for the development of CRC and can lead to intestinal injury (Zhang et al., 2017). The unclassified genus *Erysipelotrichaceae* was found to increase in cancer patients (Chen, Liu, Ling, Tong, & Xiang, 2012) and was associated with metabolic disorders and energy metabolism (Martínez et al., 2009; Turnbaugh et al., 2009). Many reports have indicated that the enrichment of *Erysipelotrichaceae* is the basis for the association between obesity and colorectal cancer (Claus et al., 2011; Zhang et al., 2009). Our results showed that alternating the use of β‐glucan and quercetin also downregulated the RA of the genus *Allobaculum* and that of the unclassified genus of *Erysipelotrichaceae*, although no significant differences were obtained. In addition, the genus *Parabacteroides* can attenuate colonic damage in azoxymethane‐treated mice (Arpaia et al., 2013). Our results showed that the RA of *Parabacteroides* was significantly higher in CRC mice that alternately consumed β‐glucan and quercetin than in mice that consumed the other diets. Hence, we inferred that the alleviation of CRC by the alternating consumption could be mainly attributed to the regulation of colon cancer‐associated bacteria.

Although the consumption of β‐glucan and quercetin was discontinuous, the intestinal environment or ecosystem was still altered, and the expression of some genes, such as *Hmgcs2*, *Fabp2*, and *Gpt*, in colonic epithelial cells was hence changed. Some studies have shown that *HMGCS2* may relate to the progression of cancer and play a key role in enhancing the phenotype of tumor migration, invasion, and metastasis through an independent metabolic mechanism in vitro. *HMGCS2* can also increase the heterotopic expression of cancer cells to enhance the vitality of cancer cells (Chen et al., 2017). Intestinal fatty acid binding protein 2 (*FABP2*) is an intracellular protein, a highly specific marker of intestinal necrosis, expressed in intestinal epithelial cells and combined with saturated and unsaturated long‐chain fatty acids to participate in the absorption and transport of dietary fatty acids (Alharbi et al., 2014; Kano, Takahashi, Inoue, Tanaka, & Okita, 2017; Khattab, Abo‐Elmatty, Ghattas, Mesbah, & Mehanna, 2017). *Gpt* is a marker of hepatocyte injury, which is higher in patients with hepatitis than in healthy people (Sosa, Vidlak, Strachota, Pavlik, & Jerrells, 2005). Our results showed that the alternating consumption downregulated these three genes, which could be responsible for the alleviation of CRC development.

However, the effect of alternating the consumption of β‐glucan and quercetin seems to be weaker than that of the continuous use of β‐glucan alone when comparing the reduced mortality with that in our previous study (12.5% vs. 30%) (Luo, Zhang, et al., 2018a). The reason may involve two factors. One could be that the consumption of the two bioactive compounds (β‐glucan and quercetin) alternated and only accounted for 2/3 of the whole experimental period. Another reason could be the appetite‐lowering effect of quercetin as an effective phytoligand for the cannabinoid‐1 (CB1) receptor, which would lower the food intake of the mice (Figure 1), probably resulting in worsening the recovery from CRC damage (Pagotto, Marsicano, Cota, Lutz, & Pasquali, 2005; Shrinivasan, Skariyachan, Aparna, & Kolte, 2012).

Taken together, the reduction of the TNF‐α level, modulation of some CRC‐associated gut bacteria, and downregulation of several CRC‐related genes were responsible for the alleviation of CRC in the mice that alternated the consumption of β‐glucan and quercetin. Therefore, alternating the consumption of β‐glucan and quercetin can contribute to host health and may even improve human health. However, this alternating consumption may weaken the positive effect of β‐glucan on CRC.

## 5 CONCLUSION

Alternating the consumption of β‐glucan and quercetin can alleviate colon injury and reduce the mortality of mice with CRC. Though the reduction was not significant, this alternating consumption regimen can protect mice against colon cancer by reducing some specific gut bacteria, such as *Allobaculum* and unclassified *Erysipelotrichaceae,* which are associated with colon cancer. Additionally, alternating the consumption of β‐glucan and quercetin can increase the abundance of *Parabacteroides,* which could alleviate CRC. Moreover, the altered intestinal ecosystem due to alternating the consumption of β‐glucan and quercetin can further result in the downregulation of 3 cancer‐associated genes (*Hmgcs2, Fabp2,* and *Gpt*). In conclusion, alternating the consumption of some bioactive compounds, such as β‐glucan and quercetin, in food would contribute to human health. This experiment provided some experimental evidence for the dietary recommendations for disease prevention and management.

## CONFLICT OF INTEREST

The authors have no conflicts of interest to declare.

## ETHICAL APPROVAL

All applicable international, national, and/or institutional guidelines for the care and use of animals were followed in this study.
